# Design of the FemCure study: prospective multicentre study on the transmission of genital and extra-genital *Chlamydia trachomatis* infections in women receiving routine care

**DOI:** 10.1186/s12879-016-1721-x

**Published:** 2016-08-08

**Authors:** Nicole H. T. M. Dukers-Muijrers, Petra F. G. Wolffs, Lisanne Eppings, Hannelore M. Götz, Sylvia M. Bruisten, Maarten F. Schim van der Loeff, Kevin Janssen, Mayk Lucchesi, Titia Heijman, Birgit H. van Benthem, Jan E. van Bergen, Servaas A. Morre, Jos Herbergs, Gerjo Kok, Mieke Steenbakkers, Arjan A. Hogewoning, Henry J. de Vries, Christian J. P. A. Hoebe

**Affiliations:** 1 Department of Sexual Health, Infectious Diseases and Environmental Health, South Limburg Public Health Service (GGD South Limburg), Geleen, The Netherlands; 2 Department of Medical Microbiology, School of Public Health and Primary Care (CAPHRI), Maastricht University Medical Centre (MUMC+), Maastricht, The Netherlands; 3 Department Infectious Disease Control, Municipal Public Health Service Rotterdam-Rijnmond (GGD Rotterdam), Rotterdam, The Netherlands; 4 National Institute of Public Health and the Environment (RIVM), Epidemiology and Surveillance Unit, Centre for Infectious Disease Control, Bilthoven, The Netherlands; 5 Department of Public Health, Erasmus MC—University Medical Center Rotterdam, Rotterdam, The Netherlands; 6 Public Health Laboratory, Public Health Service of Amsterdam (GGD Amsterdam), Amsterdam, The Netherlands; 7 Department of Infectious Diseases, Public Health Service of Amsterdam (GGD Amsterdam), Amsterdam, The Netherlands; 8 Center for Infection and Immunity Amsterdam (CINIMA), Academic Medical Center (AMC), Amsterdam, The Netherlands; 9 STI Outpatient Clinic, Public Health Service of Amsterdam (GGD Amsterdam), Amsterdam, The Netherlands; 10 Department of General Practice, Academic Medical Centre, Amsterdam, The Netherlands; 11 STI AIDS Netherlands (SOA AIDS Nederland), Amsterdam, The Netherlands; 12 Institute for Public Health Genomics (IPHG), Department of Genetics and Cell Biology, Research School GROW (School for Oncology and Developmental Biology), Faculty of Health, Medicine and Life Sciences, University of Maastricht, Maastricht, The Netherlands; 13 Department of Medical Microbiology and Infection Control, Laboratory of Immunogenetics, VU University Medical Center, Amsterdam, The Netherlands; 14 DNalysis Maastricht, Maastricht, The Netherlands; 15 Department of Work and Social Psychology, Maastricht University, Maastricht, The Netherlands; 16 Department of Dermatology, Academic Medical Center, University of Amsterdam, Amsterdam, The Netherlands

**Keywords:** Chlamydia trachomatis, Anorectal, Genital, Transmission, Heterosexual

## Abstract

**Background:**

In women, anorectal infections with *Chlamydia trachomatis* (CT) are about as common as genital CT, yet the anorectal site remains largely untested in routine care. Anorectal CT frequently co-occurs with genital CT and may thus often be treated co-incidentally. Nevertheless, post-treatment detection of CT at both anatomic sites has been demonstrated. It is unknown whether anorectal CT may play a role in post-treatment transmission. This study, called FemCure, in women who receive routine treatment (either azithromycin or doxycycline) aims to understand the post-treatment transmission of anorectal CT infections, i.e., from their male sexual partner(s) and from and to the genital region of the same woman. The secondary objective is to evaluate other reasons for CT detection by nucleic acid amplification techniques (NAAT) such as treatment failure, in order to inform guidelines to optimize CT control.

**Methods:**

A multicentre prospective cohort study (FemCure) is set up in which genital and/or anorectal CT positive women (*n* = 400) will be recruited at three large Dutch STI clinics located in South Limburg, Amsterdam and Rotterdam. The women self-collect anorectal and vaginal swabs before treatment, and at the end of weeks 1, 2, 4, 6, 8, 10, and 12. Samples are tested for presence of CT-DNA (by NAAT), load (by quantitative polymerase chain reaction -PCR), viability (by culture and viability PCR) and CT type (by multilocus sequence typing). Sexual exposure is assessed by online self-administered questionnaires and by testing samples for Y chromosomal DNA. Using logistic regression models, the impact of two key factors (i.e., sexual exposure and alternate anatomic site of infection) on detection of anorectal and genital CT will be assessed.

**Discussion:**

The FemCure study will provide insight in the role of anorectal chlamydia infection in maintaining the CT burden in the context of treatment, and it will provide practical recommendations to reduce avoidable transmission. Implications will improve care strategies that take account of anorectal CT.

**Trial registration:**

ClinicalTrials.gov Identifier: NCT02694497.

## Background

*Chlamydia trachomatis* (CT) infection is the most commonly reported sexually transmitted infection (STI) in high income countries; about 3-4 % of 18–24 year olds in the general population of European Union Member States are infected with CT [1–3]. Most infections occur in the young and the burden of sequelae is largest in women [4–6]. CT is a major cause of pelvic inflammatory disease, ectopic pregnancy and tubal infertility in women and of urethritis in men and proctitis in men who have sex with men (MSM) [4]. CT repeat infections are common in women [7], and are related to an increased risk of adverse reproductive outcomes [8]. Moreover, CT genital and anorectal infections facilitate the acquisition and transmission of HIV infection [4, 9]. Much of the burden of bacterial STI results from their frequent initial asymptomatic nature, and many infections remain undiagnosed [4]. Chlamydia is widely recognized as a public health problem and many countries have adopted control strategies to limit its spread. Measures include active testing in young people, additional population screening programs and enhanced screening activities at general practitioners [10–12]. Yet, it is clear that we fail to sufficiently limit the ongoing transmission as CT prevalence and incidence rates remain high.

Two knowledge gaps can be identified in the evidence for CT control strategies. First, we do not understand the role of anorectal CT in women in overall CT spread. In women, anorectal infections are about as common as genital infections, with proportions positive ranging between 7 - 27 % of anorectally tested women [13-25]. Yet, most anorectal CT infections remain undetected as general practitioners, hospital and population testing initiatives ignore anorectal CT and largely focus on genital CT [26]. STI clinics do test women at the anorectal site on indication, i.e., when they report anal sex or symptoms. Still, with half of all anorectal CT occurring in women who do not report anal sex, many anorectal infections are left untested. Second, we do not know whether transmission of CT can occur in the weeks following currently recommended treatment. Prior studies have shown considerable detection of both anorectal and genital CT after treatment in women and men [27–32]. Detection rates depended on the number of samples taken and type of tests used, demonstrating up to 40 % of treated cases having at least one positive sample when sampled multiple times within 8 weeks [27]. Indeed, repeat genital CT infections are common following treatment, varying between 10 % and 30 % when re-testing CT positive treated women between 3 months and a year later [33]. Guidelines advise such re-screening at 3 months after a CT diagnosis. Yet, in practice, re-screening occurs in less than a third of patients in the Netherlands [34]. Current care does not include a routine re-test of patients within 3 months of treatment [35-37] nor does it include an anorectal re-test 3 months after the genital CT treatment. Many uncertainties exist on the clinical relevance of a post-treatment CT detection. It is unknown whether it reflects transmission after sexual exposure or self-infection. It is also unknown whether detection of CT nucleic acids indicates viable or nonviable CT and what organism loads are present [38]. Suboptimal treatment may possibly play a role, but evidence is inconclusive leading to considerable debate on this issue [39–42].

The current lack of anorectal testing of patients attending STI care may not pose much of a problem when the anorectal infections are effectively treated together with the genital CT infections, as 75-95 % of anorectal CT co-occur with genital CT [13]. Yet, it is unknown whether treatment efficacy is sufficient. Moreover, the lack of understanding what observed CT positivity after treatment means in terms of transmission and morbidity, may preclude any conclusions on the potential positive effect of co-incidental anorectal CT treatment. Our understanding of how anorectal CT detection may explain CT transmission is incomplete [27]. (Un-and under) treated anorectal infections may contribute to ongoing transmission of genital and anorectal CT in the population at risk, both between partners and between anatomic sites within an individual. The FemCure study is set up to contribute to filling these knowledge gaps and contribute to the evidence for effective CT control strategies.

## Methods/design

### Study aim

The aim of the FemCure study is to understand the transmission of anorectal CT infections in women who receive routine care, specifically from their male sexual partners and to and from the anal and genital region within the same woman, and also includes an evaluation of treatment-type impact.

During a period of 4 years (2016–2019) the following study questions will be addressed:

- What is the detection rate of anorectal and genital CT in heterosexual women in the 12 weeks after receiving standard of care for a genital/anorectal CT infection?
- What is the risk of incident CT detection after sexual exposure (i.e., transmission from a male partner)?
- What is the risk of incident CT detection in alternate anatomic sites in women (i.e., transmission from one to another anatomic site in the same woman/self-infection)?

2) What are the co-factors?
3) Is treatment-type associated with risk of repeat genital/anorectal CT detection?
4) Which practical recommendations can be formulated for professionals involved in CT control (STI clinic workers, general practitioners, gynaecologists, medical microbiologists).

### Design

To reach the study aim, a multicentre prospective cohort study is set up among women visiting Dutch STI clinics.

### Setting

Women will be recruited from STI clinics of the Public Health Services (GGD) in Rotterdam, Amsterdam and South Limburg. According to our national registry these three participating clinics tested 31200 women for CT in 2014. Of these, 3718 (12 %) women tested CT positive [5]. Part of these women was also tested anorectally demonstrating about 600 anorectal CT infections. Among all CT positives, the majority was young (i.e., 71 % were under 25 years of age, 20 % were between 25 and 29 years of age). A substantial part of the women who tested CT positive had a low socio-economic status (SES), based on the SES score of their neighbourhood of residence (www.scp.nl), i.e., 21 % had low SES and 13 % had very low SES. Of the positive women, the majority was Dutch (61 %). Of the remainder of the positive women, 10 % were Surinamese, 4 % were Antillean, 3 % were Moroccan, and 1 % were Turkish. The majority of the non-Dutch ethnic groups were second generation migrants.

### Study population

The eligible study population is likely to reflect the current STI clinic populations. Eligible are heterosexual women with symptomatic or asymptomatic genital and/or anorectal CT infection, who are not pregnant and 18 years or older. Exclusion criteria are recent reported use of antibiotics, HIV positivity, syphilis and infection with *Neisseria gonorrhoea*. As single anorectal CT is uncommon and around 75-95 % of genital CT positive women also have anorectal CT [13–25], it is expected that of the included women only few will have anorectal CT only. The majority (75 %) expectedly will have anorectal CT in combination with genital CT, and most of the remainder will have genital CT only at inclusion in the study. Treatment occurs according to regular care and international guidelines [35–37]. Women who are anorectally tested in routine care and found anorectal CT positive are treated with a 7-day course of doxycycline 100 mg twice daily with the first dosis being directly observed. All other women receive a directly observed single dose of azithromycin 1000 mg [35–37]; these women tested genitally CT positive in routine care and were either anorectally untested in routine care or were tested anorectally CT negative.

### Recruitment

Participants will be recruited upon return for treatment at three STI clinics. Recruitment of women is expected to take approximately 1 year. At recruitment, the women receive information on the study and are referred to the study website: www.femcure.nl.

### Inclusion and follow-up

Participation starts after written informed consent. The women will be followed for 3 months when they collect samples and complete self-administered questionnaires (see Table 1). In order to motivate participants to complete follow-up, short text messaging (SMS) reminders are used, simple home-collection of samples is used [43, 44], and online questionnaires are used. Follow-up will entail 3 clinic visits, at weeks 4, 8, and 12. This last visit is also a routine STI clinic visit for repeat testing. Small incentives are provided (€10) at each follow-up clinic visit. Inclusion and follow-up visits are conducted by highly experienced nurses trained in STI care including motivational interviewing. Further, to increase the number of participants with complete follow-up data, the women who are included but who do not show up at week 4 for the clinic visit will be replaced by a new participant.

**Table 1:** Overview of FemCure study time points at inclusion (pre-treatment) and during follow-up until 12 weeks post-treatment

	Pre-treatment	Week 1	Week 2	Week 4	Week 6	Week 8	Week 10	Week 12
	T0 (inclusion)	T1	T2	T3	T4	T5	T6	T7
Location	Clinic	Home	Home	Clinic	Home	Clinic	Home	Clinic
Number of anorectal samples collected per woman^a^	2	1	1	2	1	2	1	2
Number of genital samples collected per woman^a^	2	1	1	2	1	2	1	2
Number of pharyngeal swabs collected per woman^b^	2			1		1		1
NAAT on self-collected swabs	X	X	X	X	X	X	X	X
Viability testing on self-collected swabs	X			X		X		X
Chlamydia organism load, MLST^c^ and semen biomarker^c^ testing on self collected swabs	X	X	X	X	X	X	X	X
(Online) short questionnaire	X	X^d^	X	X	X	X	X	X

^a^Self-collected swabs

^b^Nurse taken swabs; stored for later testing

^c^In subset only

^d^Assessing treatment compliance and sexual behavior (in short) at week 1. At the other weeks, detailed self-reported sexual behaviors and symptoms are assessed

### Sample collection

Women will collect self-administered anorectal and vaginal swabs at 8 time-points at home and at the STI clinic (see Table 1) [43, 44]. A test-package with clear instructions is provided for self-collection at home. At clinic visits, the participants take an additional self-administered anorectal and vaginal swab that is stored in a different buffer and cooled immediately at −80 °C, to allow CT viability testing. Samples are sent in batches by courier to the laboratory for processing. Each clinic visit, also a pharyngeal nurse-taken swab is collected for later testing.

### Laboratory testing

All samples are tested using routinely used nucleic acid amplification tests (NAAT) for the presence of CT (Table 1). The frozen samples from participants testing CT positive by routine NAAT are further tested for viability with viability polymerase chain reaction (vPCR; unpublished protocol, P. Wolffs, K. Janssen, N. Dukers-Muijrers, C. Hoebe, Medical Microbiology, MUMC, Maastricht) directed at the detection of DNA inside intact microbial cells or with culture [45]. A positive culture or vPCR result implies the presence of viable and infectious CT and provides information on the potential clinical relevance of the infection. Other non-routine measures include the CT-DNA load that will be assessed using quantitative NAAT. The CT genotype will be determined using high resolution multilocus sequence typing (hr-MLST) [46]. Strain typing is here specifically used to confirm self-infection or persistence. A selection of samples is tested for Y chromosome DNA as a marker for semen exposure to assess underreporting of sexual behaviour [47].

### Collection of self-reported data

Data will be collected by online self-administered questionnaires at inclusion, and during follow-up around the sampling times. The questionnaire at inclusion contains questions on age, ethnicity, gender, socio-economic status. All questionnaires contain questions on number of partners, and for each sexual partner the sexual behaviour (genital, anal, oral sex) in the past two weeks, testing for STI and treatment. It also includes questions on behaviours such as contraceptive use, antibiotic use, drug use, STI testing during follow-up, and on list of symptoms. Further, participant data will be collected concerning treatment type and compliance. For the purpose of process evaluation, a few questions are included that address acceptability and possible difficulties encountered related to the participation in the study. Each participant has a study code under which all data (samples and questionnaires) are collected and analysed.

### Outcome in analyses

Detection of anorectal and genital CT by NAAT (primary outcome), viability and load (secondary outcomes).

### Definition of transmission

Transmission is defined as an observed incident CT detection post-treatment (a) after having sex with a source partner (by self-reported behaviour and biomarker-validation), and (b) when another anatomical source location tested positive, i.e., self-infection. In such cases, self-infection is considered likely when CT strain types do not differ between anatomic sites of a woman. Samples with CT detection that follow a sample with CT detection may represent persistence and are considered not indicative for transmission. In such cases, persistence is considered likely when CT strain types do not differ in a woman over time.

### Defining sexual exposure

In women, the process of CT acquisition from an infected partner, i.e., via sex, is defined based on sexual behaviour data from the detailed and frequent questionnaires and by biomarker assessment. The actual CT status of the partner during follow-up will in most cases be unknown. Hence, the sexual behaviour that is reported during follow-up may also include sex with CT negative partners. The risk estimate for the association between sexual exposure and incident CT detection reflects the risk of transmission given this sexual practice and regardless of the partner's CT status. This will be a good proxy to the actual transmission probability for a given sexual practice in ‘real life’ and is most relevant in the practical care setting where information on sexual behaviour is routinely asked about and registered and partners’ CT status is often lacking. To validate sexual exposure from a man, especially to indicate underreporting, biomarker validation is applied.

### Defining alternate anatomic site exposure

The process of CT anorectal acquisition from her own genital CT infection and vice versa is defined based on the CT test results. Exposure is defined as presence of CT detection at the other anatomic site at the same sampling moment and/or the previous sampling moment. CT typing results are also used to confirm the link between the two detections.

### Treatment failure

A possible reason for CT detection after treatment, other than transmission, may be treatment failure. Whether antimicrobial treatment failure for CT plays a role remains poorly understood [41]. There is no evidence of homotypic anti-microbial resistance and testing for antimicrobial resistance for CT is not routinely available. It will therefore be quite difficult to microbiologically ascertain treatment failure as a reason for CT detection. Still, there is current debate on what is the appropriate treatment for anorectal CT infection [39–41]. Some studies conclude that azithromycin is a suboptimal treatment [28, 30–32], while another study found doxycycline and azithromycin treatments to be equally effective for anorectal CT [29]. In some countries (the Netherlands, Australia, UK), guidelines for anorectal CT treatment have recently shifted from azithromycin 1000 mg single dose to doxycycline 100 mg twice daily for 7 days [36, 37], while in the US these treatments are considered to be equally effective first line treatments [35]. These recommendations reflect concern that azithromycin efficacy for anorectal infections may be less than expected. Yet, there are no robust studies showing conclusive evidence concerning a suboptimal effect of any type of treatment or to prefer one over the other treatment. The current study attempts to inform this debate by analysing the association between treatment and CT detection in a sub analysis.

### Statistical analyses

The main study questions will be answered by analysing the data on the level of episodes. For each incident CT detection, the information of the preceding sample results, and the sexual behaviour information from the preceding 2-week interval, is analysed. Using mixed models, univariate and multivariate logistic regression analyses will be applied to calculate rates of incident CT anorectal and genital detection, controlling for repeated measurements in a person. Thereby, the relative contributions are assessed of sexual exposure and of alternate anatomic site exposure, i.e., sexual transmission and self-infection. This will be done by including different exposures as co-factors and this will also be done comparing categories of sexual exposure, anatomic site exposure, and both exposures compared to no such exposure (see Table 2).

**Table 2:** Main regression models in FemCure study

	Exposure categories	Definition in the data^b^	Interpretation^c^ of positive CT test result
Outcome incident genital CT detection			
Model 1	Sexual exposure^a^	Genital sexual exposure (at T_x_)	
	Alternate site exposure	Anorectal CT positive (at T_x_ and/or T_x-2_)	
Model 2	No exposure	No genital sexual exposure (at T_x_) AND anorectal CT negative (at T_x_ and T_x-2_)	Persistence
	Genital sexual exposure only	Genital sexual exposure (at T_x_) AND anorectal CT negative (at T_x_ and T_x-2_)	Sexual transmission
	Anorectal site exposure only	No genital sexual exposure (at T_x_) AND anorectal CT positive (at T_x_ and/or T_x-2_)	Transmission from anorectal site
	Sexual exposure and anorectal site exposure	Genital sexual exposure (at T_x_) AND anorectal CT positive (at T_x_ and/or T_x-2_)	Sexual transmission AND/OR Transmission from anorectal site
Outcome incident anorectal CT detection			
Model 1	Sexual exposure^a^	Anorectal sexual exposure (at T_x_)	
	Alternate site exposure	Genital CT positive (at T_x_ and/or T_x-2_)	
Model 2	No exposure	No anorectal sexual exposure (at T_x_) AND genital CT negative (at T_x_ and T_x-2_)	Persistence
	Anorectal sexual exposure only	Anorectal sexual exposure (at T_x_) AND genital CT negative (at T_x_ and T_x-2_)	Sexual transmission
	Genital site exposure only	No anorectal sexual exposure (at T_x_) AND genital CT positive (at T_x_ and/or T_x-2_)	Transmission from genital site
	Sexual exposure and genital site exposure	Anorectal sexual exposure (at T_x_) AND genital CT positive (at T_x_ and/or T_x-2_)	Sexual transmission AND/OR Transmission from genital site

^a^Based on self-administered questionnaires and semen biomarker assessment

^b^T_x:_ test result at the sampling; T_x-2:_ test result at the sampling two weeks earlier

^c^Strain typing is used to further aid interpretation of the observed positive CT test result, specifically to confirm self-infection (i.e., strain types do not differ between anatomic sites) or persistence (i.e., strain types do not differ over time)

Risks will be expressed by odds ratio‘s and 95 % confidence intervals. Co-factors (such as age, but also treatment-type) will be included in the models to account for their potential role as a confounding factor or effect-modifier in the associations between exposure and CT detection. Also, these co-factors are assessed for their association with CT detection.

Sensitivity analyses will be undertaken by varying the definition of the outcome (primary and secondary outcomes) and by varying the definition of the sexual exposure (e.g., including and excluding sexual practices). By performing a (limited) process evaluation of our study, we will obtain insights in the factors that have affected participation and possible loss-to-follow-up.

In a sub analysis, in which the unit of analysis will be an individual woman (not the 2-week episodes), a head-to-head comparison of the two treatments will be conducted.

### Numbers and power

Based on the power calculations we need to include 300 - 380 women with full retention, to be able to answer the main study question and the sub-analyses with sufficient power (at least 70 %) to show statistically significant (5 %) associations of a relevant size. This was calculated in different scenarios by varying the assumptions in number of women with full data (300–380), the percentage of CT detections (15-30 %), the percentage of reported sex during a 2-week episode (20-60 %) and the percentage of alternate anatomic site exposure (10-30 %). Accounting for women who are anorectally CT negative at inclusion, loss-to-follow up, missing data, and persistence, we need to recruit 400 women in the study to be able to have sufficient numbers of samples that can be analysed.

## Discussion

The FemCure study will deliver information on the role of anorectal CT in the transmission of CT. It may guide further care-optimization for anorectal CT testing and treatment as in current care women are not routinely tested anorectally. It will also describe the microbiological specifics of genital and anorectal CT detection after treatment, such as on the viability of detected CT, which is relevant for clinical practice and for healthcare policy making in order to reduce avoidable transmission.

### Strengths

First, this study will generate new information on the meaning and the cause of CT detection after treatment. This information goes beyond current state of the art due to its combination of different laboratory tests (NAAT, viability, load, typing) to detect CT, and by sampling both anorectal and genital anatomic sites in the same woman and multiple points in time. Second, the recruitment at multiple STI clinics, serving ample numbers of clients, allows us to study sufficient numbers of patients in a relatively short time period. Naturally, our study domain is restricted to patients consulting a STI clinic, which we consider the relevant population for this research question. We do not expect that results would be different for clients consulting a general practitioner or gynaecologist for their CT infection. Third, the study nurses and the participants are blinded to the results of the laboratory testing as testing will be done in batches afterwards. All data will be handled coded, ensuring the anonymity of included patients. Fourth, several measures are taken to optimize reliability and validity of measures of sexual exposure, including the use of pre-tested questions using standard terminologies that people have shown to comprehend, computer-assisted self-interviewing, using short recall periods (2 weeks), and using an administration mode that ensures privacy. Also self-reports are validated by applying biochemical measures. Although the Y chromosome DNA may not be detected in all exposures, if it is detected it is used to capture underreporting and instances of incorrect condom use or condom failure. These biomarker analyses will be done on already collected swabs, thus no extra swab is needed from a participant. Fifth, the research team is interdisciplinary and highly experienced in CT research and covers the different areas of expertise needed to successfully conduct the proposed research. It consists of members several Dutch Public Health Services, STI clinics, Medical Microbiological Laboratories, Universities (including behavioural science), and national institutes for policy and STI guideline development ensuring the quality of the study and the valorisation, i.e., the practical implication of results.

### Limitations

First, it cannot be ruled out that some degree of misclassification in both exposures and outcomes may occur. Sexual exposure may be underreported, even when validity and reliability of the questionnaire data are optimized and biomarker validation is applied (exposure misclassification). Also, a woman may clear the CT infection before the sampling occurs (outcome misclassification). However, an even more frequent sampling as already scheduled (at weeks 0,1,2,4,6,8, 10, and 12) was considered to potentially compromise study inclusion and retention and would also require more funds than are currently available.

Second, a randomised controlled trial (RCT) would be the most suited study design to compare efficacy of treatments, such as recently reported for genital CT [48]. The observational FemCure study is not designed as an RCT as a treatment comparison is not its main aim and an RCT would require more human and financial resources than are currently available. Nevertheless, the role of treatment can and will be evaluated in a sub-analysis by a head-to-head comparison of treatment-types. Most of the current studies reporting comparison of anorectal CT treatments similarly are observational studies with inherent biases to account for [28–32, 39–41]. By including different anatomic sites and a diverse range of laboratory and exposure measures in the current study, results will add to the existing literature and will thereby be able to inform the current debate on treatment failure.

## Abbreviations

CT, *Chlamydia trachomatis*; DNA, deoxyribonucleic acid; MLST, multilocus sequence typing; NAAT, nucleic acid amplification test; PHS, public health service; STI, sexually transmitted infection
